# PDGF-BB-derived supramolecular hydrogel for promoting skin wound healing

**DOI:** 10.1186/s12951-022-01390-0

**Published:** 2022-04-26

**Authors:** Ke Jian, Chenghao Yang, Tingting Li, Xia Wu, Jun Shen, Jiaying Wei, Zhimou Yang, Dan Yuan, Mingyi Zhao, Junfeng Shi

**Affiliations:** 1 School of Biomedical Sciences, Hunan University, Changsha, 410082 China; 2 College of Biology, Hunan University, Changsha, 410082 China; 3 Department of Pediatric, The Third Xiangya Hospital, Central South University, Changsha, 410013 China; 4 Jiangsu Center for the Collaboration and Innovation of Cancer Biotherapy, Cancer Institute, Xuzhou Medical University, Xuzhou, Jiangsu People’s Republic of China; 5 Ministry of Education, State Key Laboratory of Medicinal Chemical Biology, College of Life Sciences, Nankai University, Tianjin, 300071 People’s Republic of China

**Keywords:** PDGF-BB mimic peptide, Self-assembly, Supramolecular hydrogel, Skin repair, Bioactive peptide

## Abstract

Chronic wounds represent a major challenge to the present healthcare system. In recent decades, many topical therapies have been investigated for the treatment of chronic wounds, including different types of wound dressings, antimicrobial agents, and cell therapy. Platelet-derived growth factor (PDGF) plays an important role in wound healing and has been approved for treatment of wounds related to diabetes mellitus. However, the high cost and short retention time of PDGF protein have limited its wide application. To overcome this challenge, we designed a PDGF-mimicking peptide by connecting PDGF epitope VRKIEIVRKK and self-assembling motif derived from β-amyloid peptide. The resultant peptide can self-assemble into a fibril-rich network and leads to supramolecular hydrogelation with good stability. The hydrophilic epitope can be exposed on the surface of nanofibrils, which might contribute to the binding and activation of PDGF receptors. The forming hydrogel is able to induce the growth and migration of vascular endothelial cells and promote the formation of vascular branches. In the full-thickness skin wounds of healthy mice, after the application of the hydrogel, the density of neovascularization marked by CD31 was greater than that in the control group on Day 3. Larger collagen deposition and a thicker epidermis were observed on Day 12. These results demonstrate that the hydrogel can stimulate collagen deposition and angiogenesis, enhance skin regeneration, and show an excellent therapeutic effect. Taken together, this work not only provides new insight into the design of bioactive peptides but also offers a promising biomaterial for wound healing.

**Supplementary Information:**

The online version contains supplementary material available at 10.1186/s12951-022-01390-0.

## Introduction

Wound healing is a complicated dynamic process, and multiple events occur in an orderly and overlapping manner, including hemostasis, inflammation, proliferation, and remodelling [1]. Failure of one or several cellular processes results in poor wound healing. Recent studies revealed that the proportion of chronic wound patients in Europe is as high as 1–2%, while in the United States, approximately 3–6 million people suffer from chronic ulcers, resulting in use of substantial medical resources and patient burden [2, 3]. Although some therapies are available for wound healing, their effects are far from satisfactory, especially for treatment of chronic wounds, such as diabetic foot ulcers [4]. Growth factor therapy is promising in wound healing; it has numerous functions and promotes cell proliferation, migration, vascular formation, and other processes that are dysregulated in the process of wound repair [5–8]. Among them, platelet-derived growth factors (PDGFs) have proven effective in injury management and involve many cellular events in the healing process, including inflammatory cell recruitment, fibroblast proliferation and migration [9, 10], intraepithelial collagen deposition [5], and granulation tissue formation [11]. In 1997, a recombinant human PDGF-BB protein gel (Regranex) was approved by the FDA for the treatment of diabetic neurogenic foot ulcers [12]. However, the high cost and short lifetime of PDGFs limit their extensive applications. Thus, more efforts have been directed towards developing bioactive peptides and their derivatives that mimic the functions of PDGFs.

To activate its biological functions, PDGF monomeric proteins (PDGF-A, PDGF-B, PDGF-C, and PDGF-D) must either form homodimers or heterodimers (PDGF-AA, PDGF-BB, PDGF-AB, PDGF-CC, and PDGF-DD) and then bind with PDGF receptors (PDGFR-α and PDGFR-β) [13]. Since PDGF-BB is the best characterized member of the PDGF family [14], several groups have designed a series of peptides to modulate the interactions between PDGF-BB and its receptors. That research has resulted in the development of numerous bioactive peptides that function as antagonists or agonists, and the formation of dimers is critical for them to function [15, 16]. For example, Zamora et al. reported a PDGF agonist by connecting residues 153-162 on the PDGF-B chain (VRKIEIVRKK) to a heparin-binding sequence (RKRKLERIAR) [17]. In particular, the incorporation of cysteine residues allows the formation of a disulfide bond; thus, the resultant dimer is able to bind the PDGF receptor and exhibits biological functions.

**Fig. 1:**
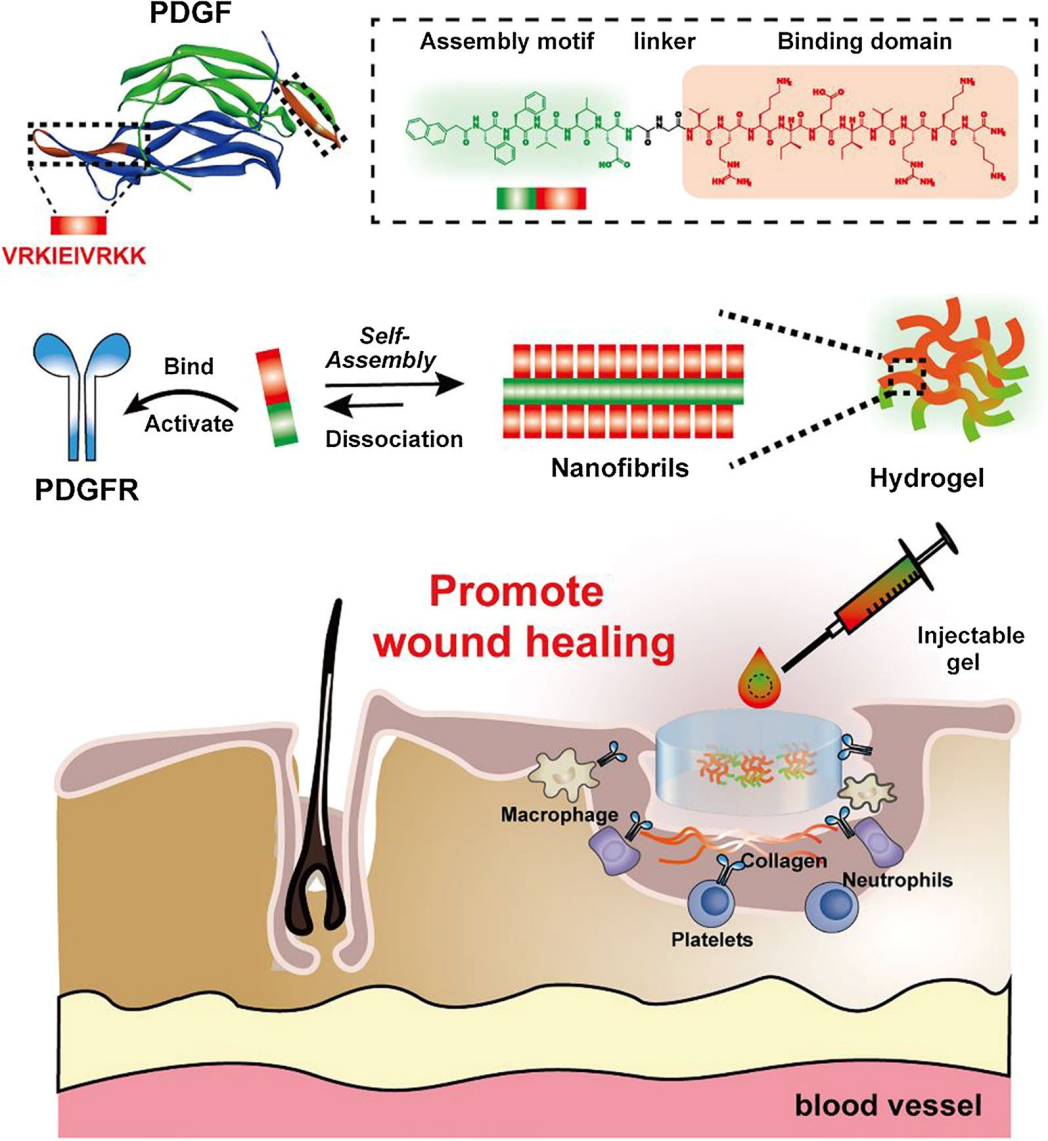
Conceptual illustration of PDGF-mimicking peptide hydrogel promotes wound healing. PDGF-mimicking peptide hydrogelators contain assembling motif, linker, and PDGFR binding domain. PDGFR binding domain is residues 153-162 displayed in orange, which binds and activates PDGF receptor. Designed peptide can self-assemble to form nanofibrils/hydrogel, which exhibits injectable properties and promotes wound healing

Supramolecular hydrogels have great advantages as wound dressings, not only because of their ability to encapsulate various bioactive agents for precise controlled release but also because they provide a moist microenvironment and absorb excess exudate to accelerate wound healing [18–20]. Driven by noncovalent interactions, small molecules can self-assemble in water to form functional entities with emergent properties [21–23]. For example, they sequester proteins [24], activate proenzymes [25], selectively inhibit cancer cells [26], and recruit mRNA to form RNA granules [27]. Notably, Yang et al., reported a PDGF-mimicking peptide by covalently linking the self-assembling motif NapFFG and the sequence VRKKP on the PDGF-B chain; this resulted in soluble nanofibers rather than hydrogel and showed a good therapeutic effect on an ionizing radiation-induced mouse model [28]. Encouraged by these pioneering works, we opted to design a PDGF-mimicking peptide by incorporating a PDGF-B active epitope to produce a new supramolecular hydrogel. As shown in Fig. 1, the designed peptide contains a PDGFR binding domain and a self-assembling motif and can thus self-assemble to form a stable hydrogel in aqueous solution. The resulting supramolecular hydrogel has the capability to enhance cell proliferation and cell migration by activating PDGF receptors. Furthermore, treating full-thickness skin wounds with PDGF-mimicking hydrogel stimulated wound healing and promoted vascularization. This hydrogel not only provides a friendly environment for the injury site but also serves as a self-delivery hydrogel depot to sustain the release of PDGF-BB mimicking peptides to promote angiogenesis and epidermal regeneration. This work is expected to help develop a new molecular therapy for wound healing.

## Results and discussion

### Molecular design

To construct a PDGF-mimicking peptide hydrogel, we envisioned the integration of a self-assembly domain and a PDGF epitope capable of activating the function of PDGFR for wound healing. Based on this rationale, we designed a peptide hydrogelator Nap-FFVLE-GG-VRKIEIVRKK (denoted as **1**). **1** contains three segments: (1) FFVLE is a β-sheet forming peptide derived from β-amyloid peptide, which has been proven to promote self-assembly [22]. The incorporation of the hydrophobic motif Nap enhances this self-assembly ability; (2) As a linker, Gly-Gly increases the flexibility of the binding domain, resulting in better accessibility of the binding domain to PDGFR; (3) VRKIEIVRKK, residues 153-162 on the PDGF-B chain, is the key domain that interacts with PDGFR, as shown in the crystal structures of PDGF-B in Fig. 1. [29] The acetyl capping PDGF epitope Ac-VRKIEIVRKK was used as a control molecule (referred to as **2**). The binding sequence is very hydrophilic in a neutral environment (five positive charges), allowing it to be exposed on the surface of the hydrophobic core of assembly **1**. Therefore, we developed a PDGF-mimicking peptide hydrogel capable of interacting with PDGFR and activating its function in wound healing. The designed molecules were synthesized via standard solid-phase peptide synthesis, purified with reverse-phase high-performance liquid chromatography (RP-HPLC), and characterized by analytical HPLC and MS spectroscopy. All data are provided in Additional file 1: Figs. S1, S2.

**Fig. 2:**
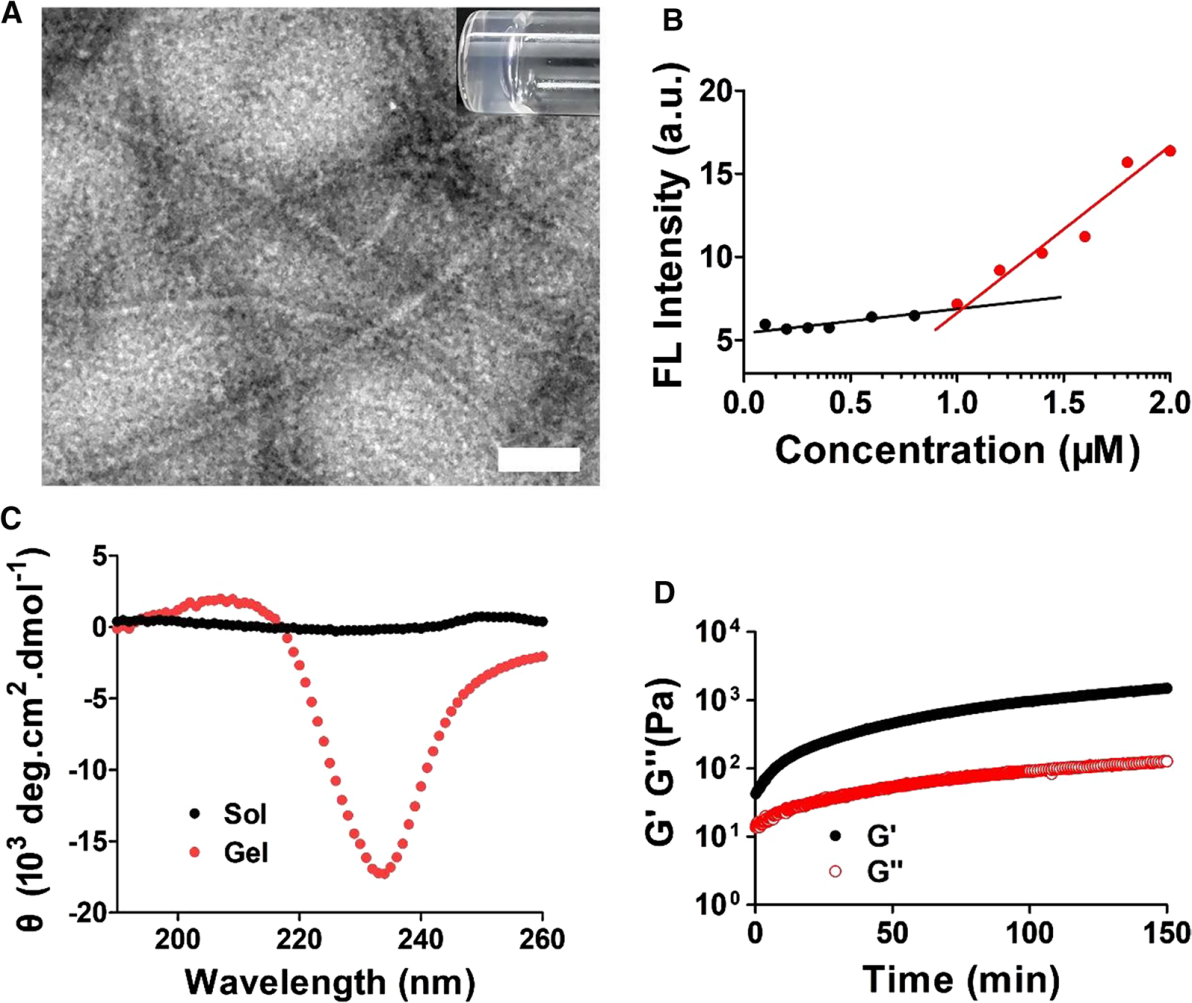
Biophysical characterization of **1**. **A** TEM image of Gel **1** at the concentration of 1.0 wt% in PBS buffer, Scale bar = 100 nm. Inset is an optical image of Gel **1**. **B** Critical aggregation concentration (CAC) of **1** determined with dye ThT. **C** CD spectra of Gel **1** (1.0 wt%, in PBS) and Sol **1** (1.0 wt% in D.I. H_2_O). **D** Rheological dynamic time sweep of Gel **1** for monitoring the storage modulus (G′) as a function of time

After obtaining these peptides, we first evaluated their self-assembly behaviours. Simple mixing of an equal volume of solution **1** (2.0 wt%) and 2X PBS buffer generates a transparent hydrogel (inset of Fig. 2A). As expected, **1** can form hydrogel when the pH was adjusted to 9.0 (boric buffer), but become solution in acidic condition (pH 5.0, Additional file 1: Fig. S3). This is due to the protonation or deprotonation of lysine and arginine side chains of peptide **1** at different pH. Conversely, control molecule **2** hardly forms a viscous fluid at a concentration of 1.0 wt%. These results indicated that NapFFVLE is an excellent self-assembling motif to trigger the gelation of a hydrophilic molecule. As shown in the transmission electron microscope (TEM) image in Fig. 2A, **1** self-assembled to form nanofibers with widths of approximately 15 nm, which is twice the length of **1**. Based on this observation, we proposed plausible molecular arrangement of **1** in the fibril (Additional file 1: Fig. S4). These long nanofibers were intertwined with each other to form a 3-dimensional network, holding a large amount of water and resulting in hydrogelation. To evaluate the self-assembling ability of **1**, we measured its critical aggregation concentration (CAC) by using thioflavin T (ThT). Figure 2B shows that the CAC value of **1** was approximately 1.0 μM, further verifying its exceptional self-assembling ability. The CD spectra revealed that gel **1** has a positive peak at 211 nm and a negative peak at 238 nm (Fig. 2C), suggesting the formation of a β-sheet-like structure. This is consistent with the fact that the natural PDGF-BB protein also forms a β-sheet conformation [30]. Thus, assembly **1** is able to mimic PDGF’s conformation, assuming that this is the structural basis for simulating the biological activity of PDGF-BB. Additionally, the CD spectrum of Sol **1** only exhibited a positive peak at 242 nm, indicating the random coil structure of **1** before self-assembly (Fig. 2C). Rheological time sweep revealed the kinetics of supramolecular hydrogelation, in which the storage modulus increased gradually and reached 1500 Pa, suggesting that **1** forms a robust hydrogel (Fig. 2D). It is well known that wound dressings should maintain their integrity to protect the wound, and Gel **1** has sufficient strength to provide adequate protection to the wound.

### PDGF-mimicking peptide promotes cell proliferation, migration and angiogenesis in vitro

**Fig. 3:**
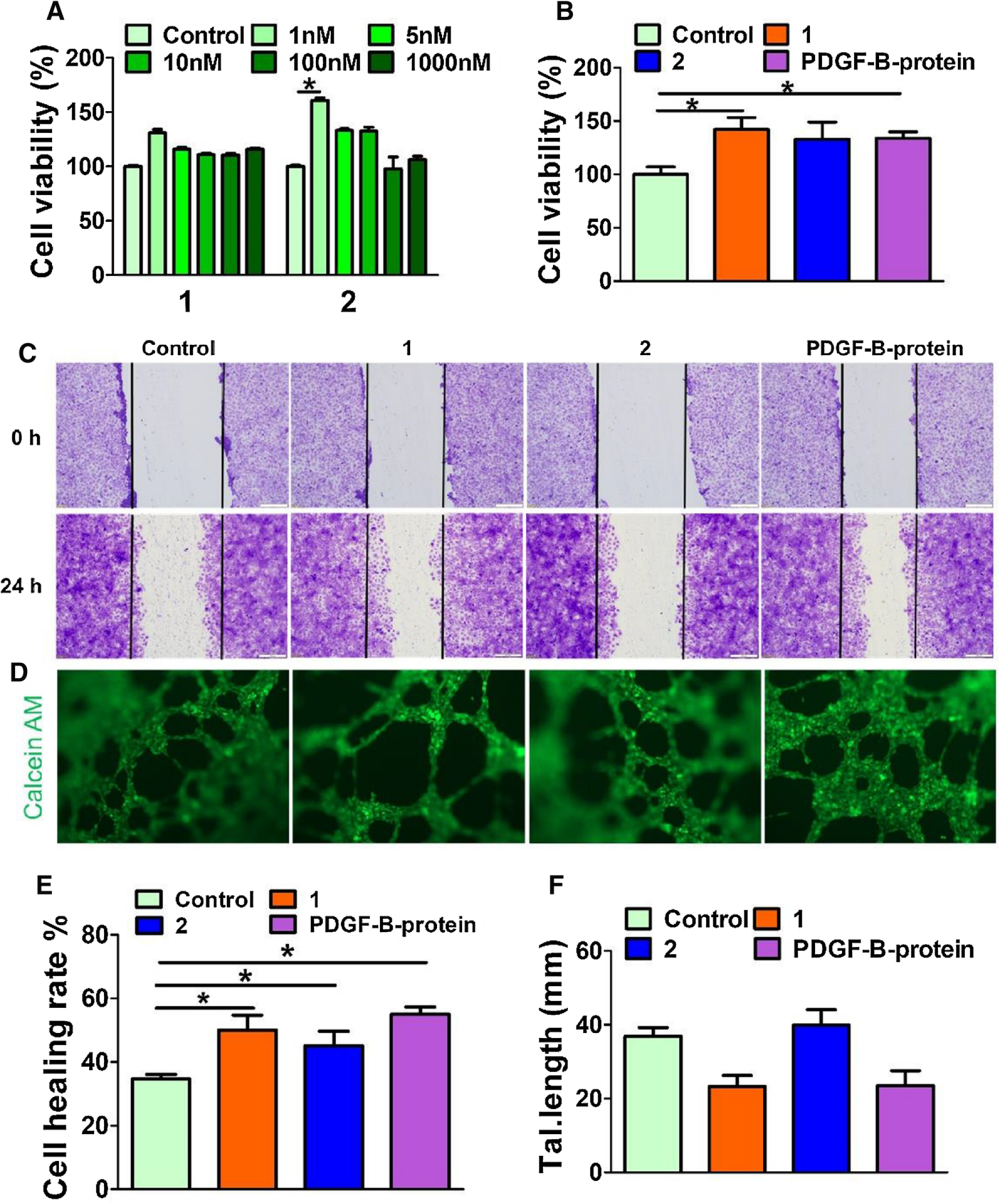
Bioactivity of PDGF mimicking peptides. **A** Cell viability of HUVEC cells incubated with **1** and **2** for 24 h. **B** Cell viability of HUVEC cells in serum-free culture medium containing 1 nM of **1**, **2** or PDGF-B protein for 24 h. Data presented as the mean ± SEM, n = 3 samples per group. **C** Representative images of HUVECs after treated with **1**, **2** or PDGF-B protein (1 nM) for 24 h. The edge of bilateral cell migration was marked with a black line. Image was taken at 10× magnification, scale bar = 100 μm. **D** Microvessel formation assay, HUVECs incubated with **1**, **2** or PDGF-B protein (1 nM) for 6 h, then stained with Calcein AM. Scale bar = 100 μm. **E** Quantification of HUVECs cell healing rate, which was determined by image J. **F** The counts of branching interval quantification of HUVECs was determined by image J. Angiogenesis.*p < 0.05 V.S. control group

In the injured area, PDGF-BB is an effective mitogen that stimulates the proliferation of fibroblasts and keratinocytes, primarily vascular endothelial cells. It also stimulates macrophages to activate and secrete growth factors, such as TGF-β [20]. Therefore, we hypothesized that molecule **1** also has the ability to stimulate wound cell proliferation. Thus, we evaluated the capabilities of molecules **1** and **2** to promote cell proliferation. First, primary human umbilical vein endothelial cells (HUVECs) were treated with different concentrations (1–1000 nM) of self-assembling peptide **1** and peptide **2** (Ac-VRKIEIVRKK, PDGFR binding domain). As shown in Fig. 3A, both **1** and **2** can stimulate cell proliferation at the measured concentration, and the optimal concentration was approximately 1.0 nM, which is comparable with PDGF protein (Additional file 1: Fig. S6). Notably, **1** and **2** displayed slightly difference on stimulating cell proliferation, we speculate that the incorporation of assembling motif still has influence on binding events between PDGF epitope and its receptor, such influence might originate from sequence itself rather than assembling events. Interestingly, **1** still exhibited the capability to enhance cell proliferation even at concentrations as high as 1000 nM. Since serum protein is an essential component for cell growth in culture and might affect the activity of the designed peptide, it is more meaningful to evaluate the functions of PDGF-mimicking peptides in the absence of serum protein. Therefore, we assessed the abilities of peptide **1**–**2** to stimulate HUVEC proliferation in serum-free culture medium for 24 h. At a concentration of 1.0 nM, **1** showed a significant proliferation-promoting effect compared with the blank. The effect was similar to that of PDGF-B protein and **2**, indicating that both **1** and **2** had PDGF-B protein-like biological activity (Fig. 3B).

The migration of skin cells is very important for wound healing. Previous reports have demonstrated that PDGF protein can increase cell migration to promote wound healing [31]. We used a scratch migration assay to study the effects of **1** and **2** on HUVEC migration. HUVECs were incubated with **1**, **2,** and PDGF-B protein at a concentration of 1.0 nM for 24 h. It is clear that peptide **1** increased the cell migration rate significantly; this increase was slightly higher than that of **2** and comparable to that of PDGF-B protein (Fig. 3C, E). This result further proved that peptide **1** can mimic the function of PDGF-B protein to promote cell migration.

Microvessel formation plays a vital role in wound healing and tissue regeneration [32]; thus, we examined the effect of PDGF-mimicking peptides on angiogenesis. Similar to the untreated group, the capillary network treated with **2** looked disorganized, finer, and discontinuous. Tissues stimulated by **1** and PDGF-B proteins formed complex, highly branched capillary-like structures, with increased branching and cross-linking of blood vessels. Quantifying the distance between the branch points along the blood vessels (called the “branch interval”), **1** and PDGF-B proteins showed reduced branching intervals, indicating a significant increase in driven vascular branches (Fig. 3F). In contrast, there was no significant difference in branch interval between Group **2** and the untreated group, likely because peptide **1** self-assembles to form a β-sheet structure. Taken together, these results showed that **1** has great potential in wound healing; both **1** and **2** can promote cell proliferation and migration, but molecule **1** is superior to **2** in stimulating HUVECs to form more vascular branches.

### Wound healing and angiogenesis in vivo

**Fig. 4:**
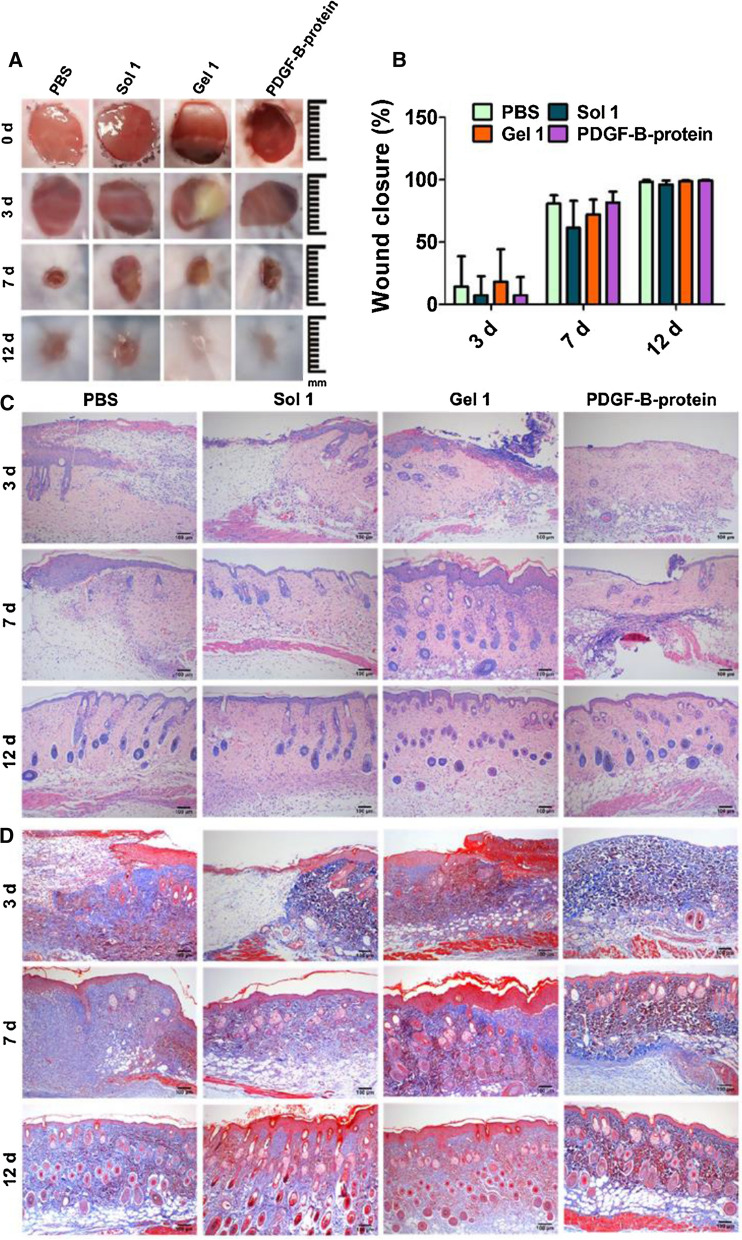
**A** Photographic images of wounds on the skin of mice treated with PBS, Sol **1**, Gel **1** and PDGF-B protein on Day 0, 3, 7 and 12. **B** Quantification of wound closure rate in mouse after different treatments. **C** H&E staining for skin tissues collected from different groups and **D** Masson’s trichrome staining for the deposition of tissue matrices at the site of injury in different groups at Day 3, Day 7 and Day 12. n = 6 mice per group

To investigate the efficacy of Gel **1** on wound healing, we established a full-thickness skin wound mouse model. Gel **1** was applied locally on the wound surface once during the whole experiment, and the wound area was monitored as a function of time. PBS, Sol **1**, and PDGF-B were also used for comparison. It is clear that the wound area of each group decreased significantly at Day 7, and all the wounds had basically healed at Day 12 (Fig. 4A). During the wound healing process, all mice were still alive, and no adverse effects were observed. Notably, no significant differences were observed between treatment groups, and all mice healed after 12 days (Fig. 4B). In contrast, the new epidermis of the Gel **1** group was thicker than that of the other three groups, implying that Gel **1** increases epithelialization because it can be slowly released to promote cell proliferation.

To further assess the efficacy of Gel **1** on wound healing, we collected the tissues around the wound and stained them with H&E (Fig. 4C). Compared with the PBS group, more neovascularization was observed in Gel **1** after 3 days. The regenerated epidermis was observed on Day 7, and the epidermis in all groups was completely remodelled on Day 12. Additionally, the regenerated epidermis in the Gel **1** group was thicker than that in PBS. Furthermore, a more regenerated papillary layer, sebaceous glands, and other accessory organs were observed. Therefore, Gel **1** accelerated the epithelialization of regenerated tissue and exhibited a better therapeutic effect. Collagen deposition during wound healing was examined using Masson staining (Fig. 4D). The amount of collagen increased gradually in each group during the experiment. In the PBS group, only a few collagen bundles were formed and arranged loosely. The Gel **1** group had larger collagen deposition areas, indicating that Gel **1** promoted collagen deposition in the wound site. Unlike the PBS group, the collagen fibers were much clearer and more organized. Since blood vessels transport oxygen and nutrients to the wound tissue and play a key role in wound healing [33], the expression level of the vascular endothelial-specific marker CD31 was examined [34]. Immunohistochemistry staining (Additional file 1: Fig. S7) on Day 3 showed that the density of neovascularization in the Gel **1** group was significantly higher than that in the PBS group and even higher than that in the Sol **1** and PDGF-B groups. These results indicated that Gel **1** provides a favourable microenvironment and stimulates angiogenesis for wound healing. Ideally, Gel **1** can slowly dissociate to release **1**, prolong its action time to match the wound healing period and stimulate wound healing. However, it is worth noting that the wound healing rate of normal mice is high, and PDGF-B is unable to accelerate this process. In addition, a full-thickness skin wound mouse model with simple resection may not be the best model because of its great limitations. Unlike humans, contraction is significant in the skin healing process of rodents, which accelerates wound closure [35]. This may be one of the reasons why there was no significant difference in the wound healing rate in vivo. Wang’s splint wound model can limit wound closure caused by skin contraction, thus simulating the healing process of human granulation growth and re-epithelialization and reducing differences in in vivo experiments [36]. Therefore, studying the effect of Gel **1** in a mouse splint model or chronic wound models, such as diabetic wounds [37] or burn wound models, would be worthwhile [38].

## Conclusion

Because of the long period and high cost, chronic wounds pose a major economic problem to society. New drugs and methods to effectively stimulate wound healing are urgently needed. Platelet-derived growth factor (PDGF) is an important growth factor secreted by cells in the wound site that affects the process of wound healing in many aspects, such as angiogenesis, regulation of inflammation, stimulation of cell proliferation and migration, collagen deposition and so on. However, protein drugs have their own challenges, such as low stability, high cost, and short action time. The development of PDGF-mimicking peptides may overcome this problem. Herein, we report a novel bioactive peptide hydrogel by combining the PDGF epitope VRKIEIVRKK and the self-assembling peptide Nap-FFVLE, which exhibits superior therapeutic effects and accelerated wound healing. Specifically, Gel **1** has the ability to induce the growth and migration of vascular endothelial cells and promote the formation of vascular branches. The application of Gel **1** in the full-thickness skin wounds of healthy mice stimulated collagen deposition and angiogenesis and enhanced skin regeneration. This work provides a new strategy to design bioactive peptides for mimicking functional proteins and generates a promising biomaterial to replace PDGF proteins in wound healing. Obviously, this strategy can be expanded to design other growth factor-mimicking peptides, such as neurotrophin-3[39].

## Supplementary Information

**Additional file 1: Figure S1.**(A) Chemical structure of 1. (B) Analytical HPLC (C) Mass spectra of 1. **Figure S2.** (A) Chemical structure of 2. (B) Analytical HPLC (C) Mass spectra of 2. **Figure S3.** Optical images of 1 prepared in (A) pH 9.0 boric buffer and (B) pH 5.0 PBS buffer. **Figure S4.** Plausible molecular arrangement of 1 in the fibril. **Figure S5.** Rheological dynamic frequency sweep test of Gel 1. **Figure S6.** Cell viability of HUVEC cells incubated with PDGF-B for 24 h. **Figure S7.** (A) Immunohistochemistry staining for CD31 of wounds in different groups on day 3. Scale bar = 100 μm. (B) The quantification of capillary on day 3. Angiogenesis of wounds was determined by image J. *p < 0.05 v.s. control group. n = 3 mice per group.

## Data Availability

The data in this work are available in the manuscript or Additional file or available from the corresponding author upon reasonable request.

## Abbreviations

PDGF: Platelet-derived growth factor
PBS: Phosphate buffered solution
RP-HPLC: Reverse-phase high-performance liquid chromatography
CAC: Critical aggregation concentration
TEM: Transmission electron microscope
CAC: Critical aggregation concentration
ThT: Thioflavin T
HUVEC: Human umbilical vein endothelial cell
