# Supplementary material for: PDGF-BB-derived supramolecular hydrogel for promoting skin wound healing
Source: J Nanobiotechnology. 2022 Apr 26;20:201. doi: 10.1186/s12951-022-01390-0 (PMC9044828; doi:10.1186/s12951-022-01390-0)
Supplement: Supplementary file 1 — Additional file 1: Figure S1.(A) Chemical structure of 1. (B) Analytical HPLC (C) Mass spectra of 1. Figure S2. (A) Chemical structure of 2. (B) Analytical HPLC (C) Mass spectra of 2. Figure S3. Optical images of 1 prepared in (A) pH 9.0 boric buffer and (B) pH 5.0 PBS buffer. Figure S4. Plausible molecular arrangement of 1 in the fibril. Figure S5. Rheological dynamic frequency sweep test of Gel 1. Figure S6. Cell viability of HUVEC cells incubated with PDGF-B for 24 h. Figure S7. (A) Immunohistochemistry staining for CD31 of wounds in different groups on day 3. Scale bar = 100 μm. (B) The quantification of capillary on day 3. Angiogenesis of wounds was determined by image J. *p < 0.05 v.s. control group. n = 3 mice per group. [file 12951_2022_1390_MOESM1_ESM.docx]

**Additional file 1**

**PDGF-BB Derived Supramolecular Hydrogel for Promoting Skin Wound Healing**

**Table of Contents**

**Table S1:** Peptides studied in this work.

**Figure S1:** Chemical structure, analytical HPLC and MS spectra of **1**.

**Figure S2:** Chemical structure, analytical HPLC and MS spectra of **2**.

**Figure S3:** Optical images of **1** prepared in (A) pH 9.0 boric buffer and (B) pH 5.0 PBS buffer.

**Figure S4:** Plausible molecular arrangement of **1** in the fibril.

**Figure S5:** Rheological dynamic frequency sweep test of Gel **1**.

**Figure S6:** Cell viability of HUVEC cells incubated with PDGF-B for 24 h.

**Figure S7:** Immunohistochemistry staining for CD31 on day 3 and the counts of capillary quantification.

**Materials and Methods**

**Table S1.** A list of reagents used in this work

| Name | [Brand](javascript:;) | Cat |
| --- | --- | --- |
| FBS | Gibco | 10270106 |
| DMEM | Gibco | C11995500BT |
| PDGF-B protein | Sino Biological | 10572-H07Y |
| Amino acid | CSBio | RBB009 |
| DMF | Meryer | M33593 |
| HBTU | Aladdin | [H106174](https://www.aladdin-e.com/zh_cn/h106174.html) |
| AM resins | CSBio | RAJ001 |
| TFA | Macklin | T818778 |
| CCK-8 | [Topscience](http://www.baidu.com/link?url=1Hy_XnsIc7TqK9JegNE_y_RCApTNjWkuO-8CVn0Xw12eOJwJKh754hNoKCe5LR3N) | C0005 |
| Matrigel | BD, Cornig | 356230 |
| Calcine AM | Beyotime | C2012 |

**Table S2.** A list of equipment used in this work

| Name | Model |
| --- | --- |
| Peptide synthesizer | CS136S |
| TEM | JEM-2100plus |
| Rheometer | MCR-92 |
| HPLC | NS4205 |
| CD | Jasco-815 |
| Inverted fluorescence microscope | IX-73 |
| MS | UltrafleXtreme |
| UV spectrophotometer | UV-6100A |

**Peptide Synthesis**

All peptides were synthesized via standard solid phase peptide synthesis (SPPS) using CSBio peptide synthesizer, with AM resins and activation by HCTU. The peptide was cleaved from the resin with a cocktail of TFA/Tis/water (95: 2.5: 2.5) for 3 h under a nitrogen atmosphere. The resin mixture was filtered and washed with excess TFA. Adding cold ether into filtered solution, the crude product was obtained by using centrifugation at 3000 rpm for 5 min. The obtained crude product was dried by vacuum pump and purified by high performance liquid chromatography. All peptides were lyophilized after purification, then analyzed using analytical HPLC and MS.

**Preparation of Hydrogel**

In a typical experiment, 2 mg of **1** dissolved in 100 μL D.I. water, then added 100 μL 2x PBS (pH 7.4, 274 mM NaCl, 5.4mM KCl, 20mM Na_2_HPO_4_, 4mM KH_2_PO_4_) into the solution **1**. The suspension was stored in an incubator overnight at 37 ℃ to afford a hydrogel at the concentration of 1.0 wt%.

**Transmission Electron Microscopy**

In a typical experiment, 5 μL of hydrogel 1 was placed on carbon-coated copper grid and allowed to stand for 1 min, which was blotted by filter paper. Then the samples stained by 10 μL of uranium acetate (1 wt%) and allowed stand for 1-2 min, then blotted with filter paper, subsequently washed with a drop of water three times and then left to air dry. At last, the sample was subject to the TEM for imaging.

**Critical aggregation concentration (CAC) measurements**

In a typical experiment, a series of solution **1** at different concentrations was prepared in the pH 7.4 PBS buffer. After incubating with thioflavine T (ThT, 20 μM), the fluorescence spectra were recorded between 465 to 540 nm (λ_ex_=440nm).

**Circular dichroism (CD) spectrum**

CD spectra were collected on a Jasco J-1500 spectrometer and sample measurement with 0.1 cm quartz cell. The hydrogels were prepared in physiological buffer and stabilized at 37 ℃ incubator for 2h. Wavelength were then scanned at room temperature between 190 and 280 nm at 1 nm intervals. The data were processed using GraphPad Prism 5. Mean residue ellipticity [θ] was calculated using the following equation [θ] = (θ_obs_/10lc)/r, where θ_obs_ is the observed ellipticity in millidegrees, l is the length of the cell (cm), c is the concentration (M), and r is the number of residues.

**Oscillatory Rheology**

Rheological properties of hydrogel was evaluated by an Anton-Paar MCR 92 rheometer (Anton Paar, Graz, Austria) using a parallel plate geometry (25 mm plate diameter, 0.5 mm sample gap). In a typical time-dependent experiment, 100 μL of hydrogel **1** was transferred to the plate and geometry was lowered to 0.5 mm, after which the modulus was a monitor at the frequency of 6 rad/s and the constant strain of 0.2%. To avoid dehydration, a layer of standard silicon oil was used around the edge of sample at the beginning of experiment. Dynamic frequency and strain sweep experiments were performed to ensure that the time-sweep data were collected in the linear regime of strain and frequency. Dynamic frequency sweep was carried out in the frequency range from 0.1 to 100 rad/s at the constant strain (0.2%) after the time sweep. All rheology of gels were tested at 37℃.

**Cell Viability Assay**

All the cell lines used in this work were cultured in basic medium supplemented with 10% fetal bovine serum. For cytotoxicity measurement, cells were seeded at a density of 5000 cells/well in 96-well plates and cultured overnight. Then work solution of component **1** and **2** were diluted in FBS-free medium at different concentration of 0 nM, 1 nM, 5 nM, 10 nM, 100 nM, 1000 nM. Then the cells were incubated with work solution, and the cell viability was determined by the CCK-8 kit after 24 h. PDGF-B protein served as the positive control.

**Scratch wound migration assay**

The migration ability of HUVEC was evaluated by scratch migration assay. The cells were implanted in 6-well plates with a density of 5x10^5^ cells/well. When cells confluence reached over 90%, a scratch wound was generated in the cell monolayers with a sterile plastic 10 μL micropipette tip. Then the cells were incubated with peptide solution for 24 h. The cells were fixed with 4% paraformaldehyde for 10 min and washed with PBS for three times, subsequently stained with 0.1% crystal violet for 10 min. Photographs were captured with fluorescence microscope. The scratch area was measured using Image J software.

**Tube formation**

The tips and 96-well plates were pre-cooled before use. In brief, 25 μL of matrigel was seeded into a 96-well plate in a cold environment and incubated at 37 ℃ for 30 min. Then 1.5 ×10^4^ HUVECs cells/well were mixed with peptide solution and incubated for 6 h. The cells were stained by 5 μM calcein AM for 30 min and then gently washed with PBS. The images of tube formation were captured with fluorescence microscope and the angiogenesis was analyzed by Image J.

**Full-thickness wound model**

The animal protocols were approved by the Animal Care and Use Committee of Hunan University and conformed to the Guidelines for the Care and Use of Laboratory Animals published by the National Institutes of Health. C57BL/6 mice (male, 18-20 g) were provided by the Hunan SJA Laboratory Animal Co., Ltd (Changsha, China). 30 male mice were anesthetized by 2.5% pentobarbital, shaved, and then two full-thickness (6 mm diameter, 1 mm depth) round excisional wounds were made on the back of each mouse. The mice were randomly divided into four groups: i) blank control (no treatment except for PBS), ii) Sol **1**, iii) Gel **1**. iv) PDGF-B protein. All of drugs were injected and spread onto the wound site evenly (100 μL per site). To maintain wound clean, the wound site was covered by sterile gauzes. After 0, 3, 7, and 12 days’ treatment, optical images of wound site were record and analyzed using Image J software for determined the healing rates. The healing rates of wound was calculated using the equation: healing rate (%) = (A0 [initial wound sarea] − At [residual wound area at each time point]) / (A0 [initial wound area] × 100%).

**Histology assay**

All Mice were sacrificed at day 12 to harvest the skin tissues at the wound site. The tissues samples were fixed in 4% paraformaldehyde, embedded in paraffin and cut into 5 μm sections. The section of wound sites at different time was subjected to hematoxylin and eosin staining (H&E) and Masson’s trichrome staining. For immunohistochemically staining, the sections were deparaffinized, rehydrated, and rehydrated and heated in a microwave in citrate buffer (0.01 M; pH 6.0) for 15 min to retrieve the antigen. Then the sections were treated with H_2_O_2_ and carbinol for blocking the endogenous peroxidase activity, subsequently incubated with the primary antibodies of rabbit anti-CD31 at 4 ℃ (#77699, 1:200, CST) overnight. HRP secondary antibodies (1:400, ZSGB-BIO) incubated for 30 min were used to visualize the primary antibodies. The sections were finally imaged by optical microscope (BA210T, Motic). Vessel densities were quantified using Image J. from at least three random visual fields per section.

**Statistics**

For all the quantitative statistics, at least three biological replicates were performed. Data are presented as mean ± standard deviation (SD). Statistical analyses were performed using one-way ANOVA (GraphPad Prism 5), with * standing for p < 0.05.

**Table S3** The peptide sequences used in this work

| **Name** | **Peptide Sequence** | **Molecular weight** |
| --- | --- | --- |
| **1** | Nap-FFVLE-GG-VRKIEIVRKK | 2186.68 |
| **2** | Ac-VRKIEIVRKK | 1309.67 |


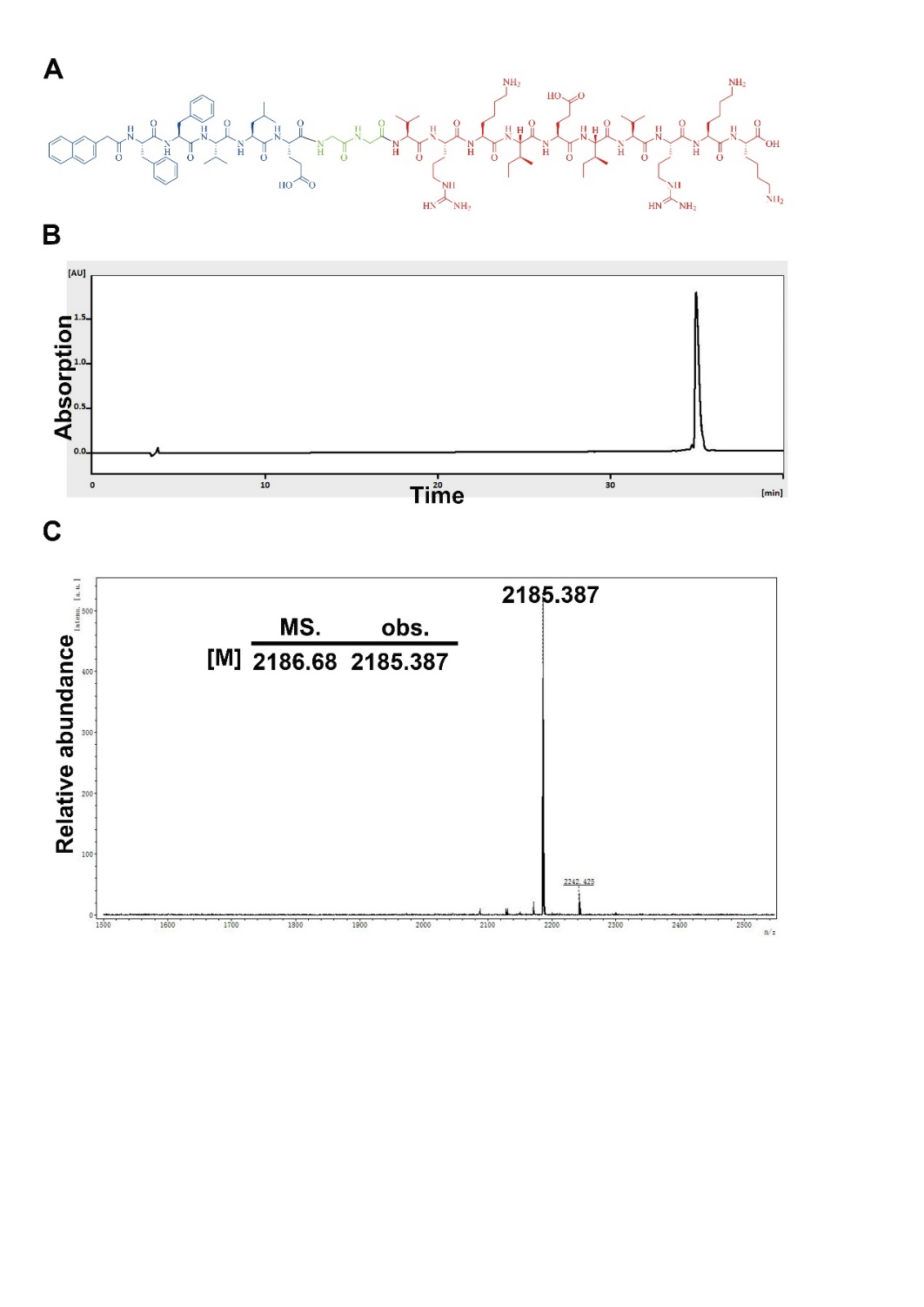


**Figure S1.** (A) Chemical structure of **1**. (B) Analytical HPLC (C) Mass spectra of **1**.


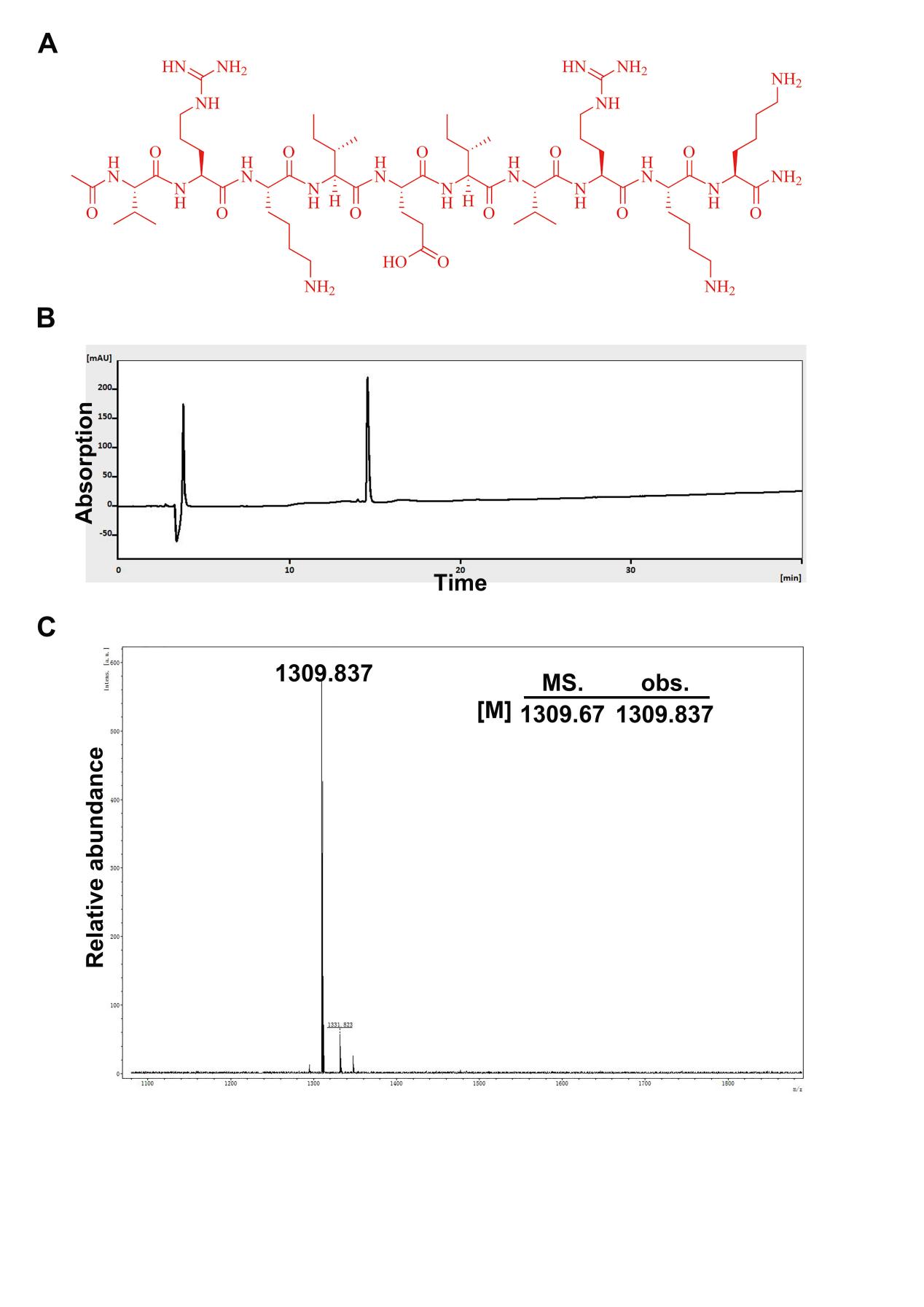


**Figure S2.** (A) Chemical structure of **2**. (B) Analytical HPLC (C) Mass spectra of **2**.


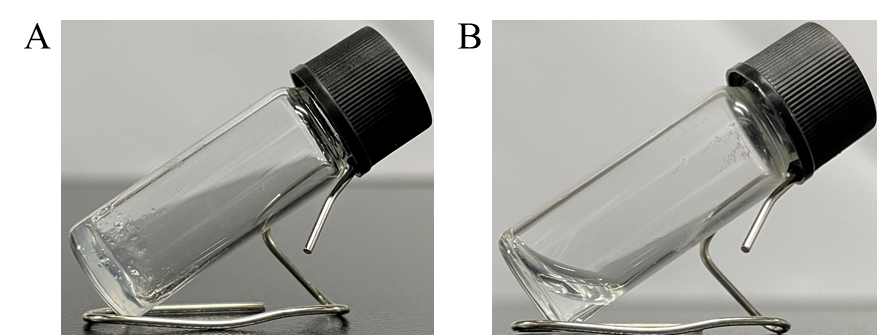


**Figure S3:** Optical images of 1 prepared in (A) pH 9.0 boric buffer and (B) pH 5.0 PBS buffer.

**
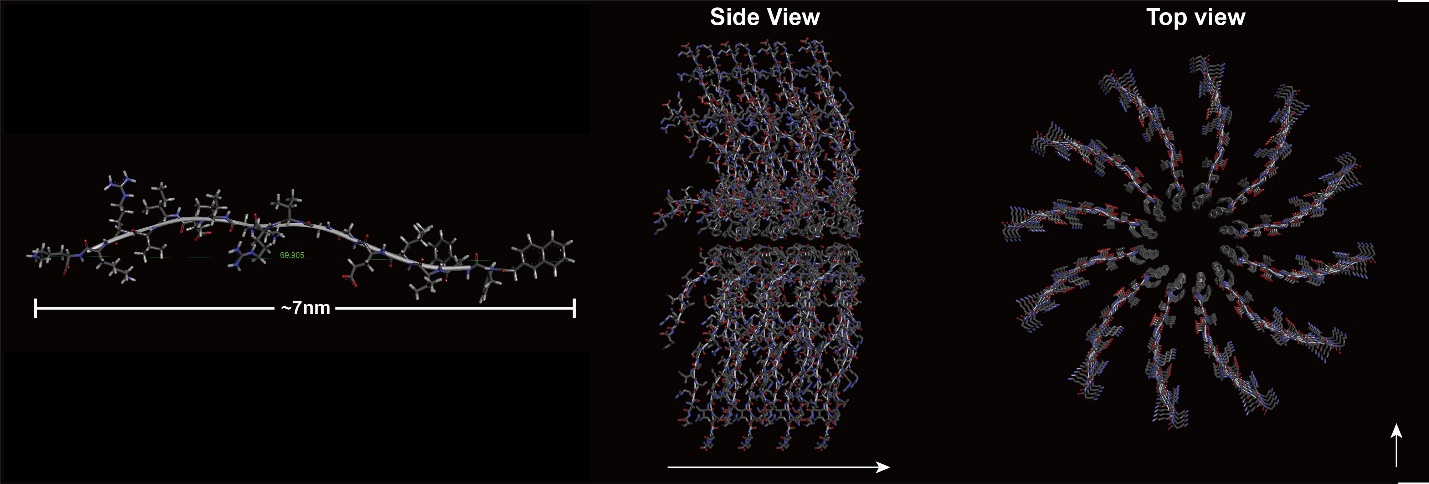
**

**Figure S4.** Plausible molecular arrangement of **1** in the fibril.


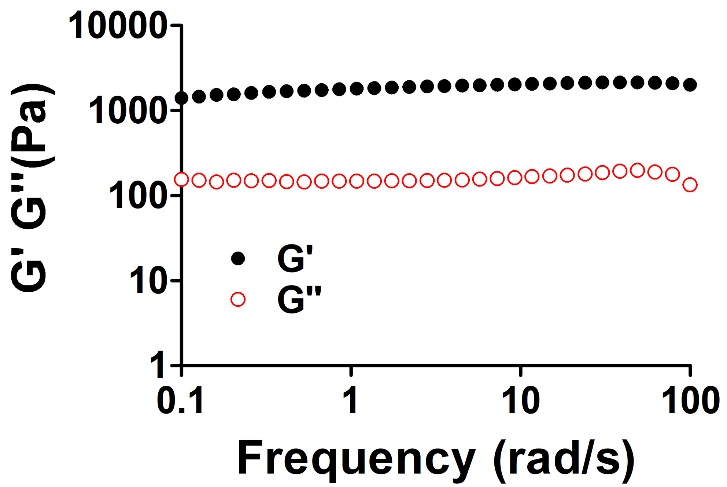


**Figure S5.** Rheological dynamic frequency sweep test of Gel **1**.

**
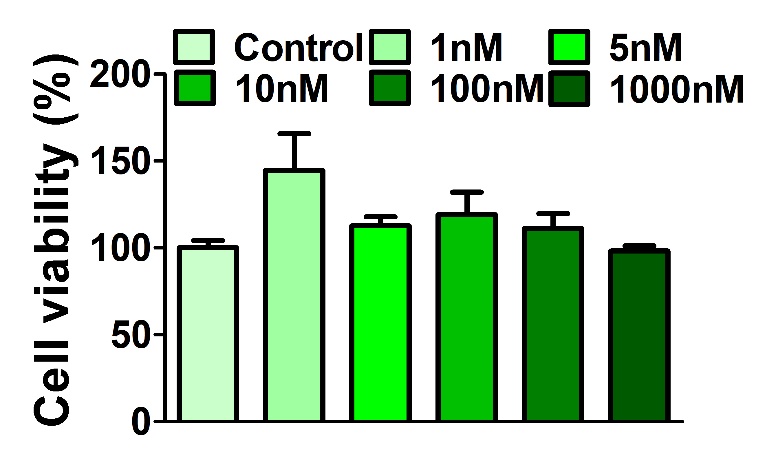
**

**Figure S6.** Cell viability of HUVEC cells incubated with **PDGF-B** for 24 h.


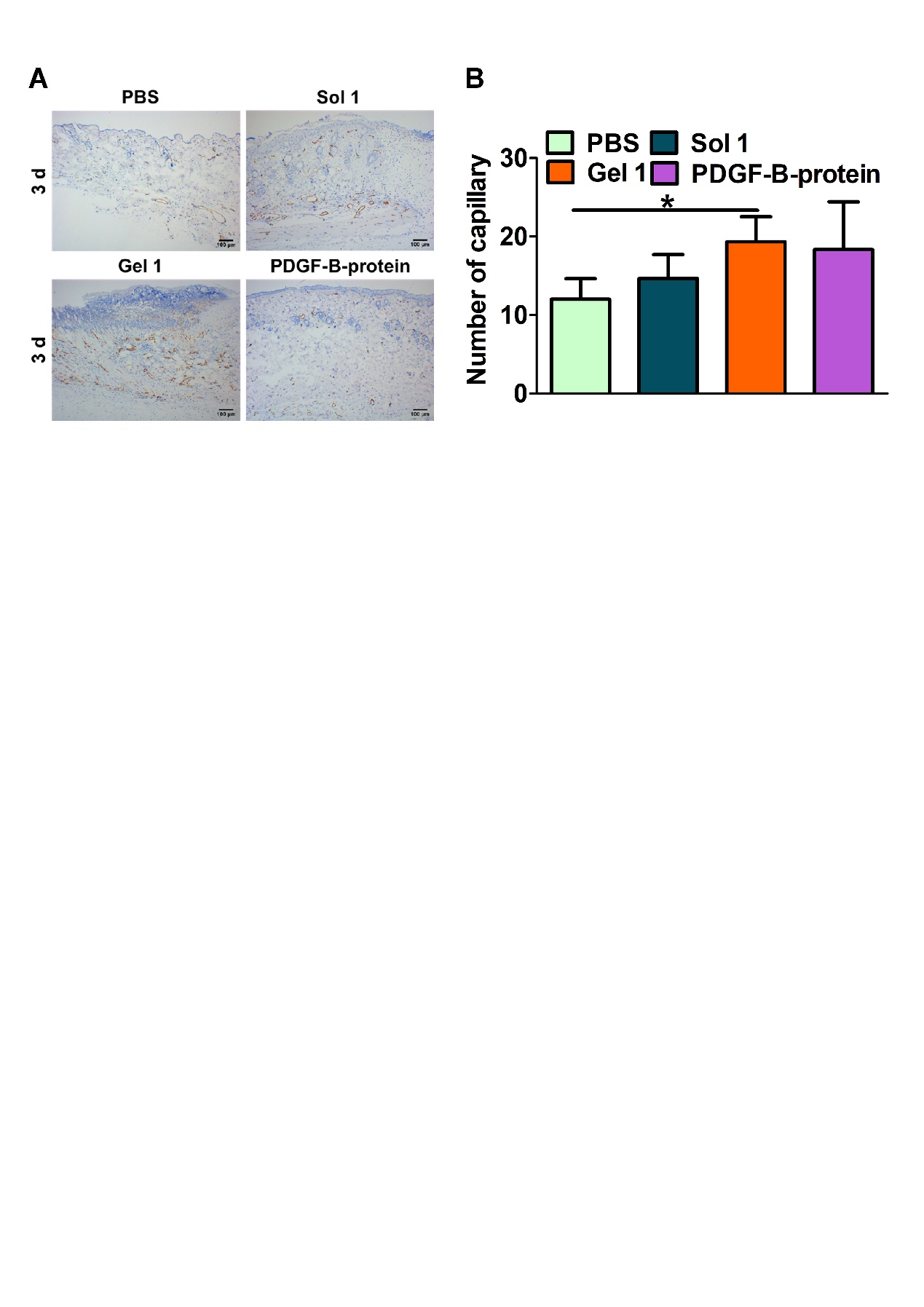


**Figure S7.** (A) Immunohistochemistry staining for CD31 of wounds in different groups on day 3. Scale bar = 100 μm. (B) The quantification of capillary on day 3. Angiogenesis of wounds was determined by image J. *p < 0.05 v.s. control group. n = 3 mice per group.
